# Glycocalyx Degradation in Pediatric Patients with Cyanotic and Acyanotic Congenital Heart Disease Undergoing Cardiac Repair Surgery

**DOI:** 10.3390/jcm15020839

**Published:** 2026-01-20

**Authors:** Judith Schiefer, Paul Lichtenegger, Eva Base, Pierre Raeven, Akos Tiboldi, Daniel Zimpfer, Erhan Urganci, Peter Faybik

**Affiliations:** 1 Department of Anaesthesia, Intensive Care Medicine and Pain Medicine, Division of General Anaesthesia and Intensive Care Medicine, Medical University of Vienna, 1090 Vienna, Austria; paul.lichtenegger@meduniwien.ac.at (P.L.); pierre.raeven@meduniwien.ac.at (P.R.); peter.faybik@meduniwien.ac.at (P.F.); 2 Department of Anaesthesia, Intensive Care Medicine and Pain Medicine, Division of Cardiac Thoracic Vascular Anesthesia and Intensive Care Medicine, Medical University of Vienna, 1090 Vienna, Austria; eva.base@meduniwien.ac.at; 3 Department of Cardiac and Thoracic Aortic Surgery, Medical University of Vienna, 1090 Vienna, Austria; daniel.zimpfer@meduniwien.ac.at (D.Z.); erhan.urganci@meduniwien.ac.at (E.U.)

**Keywords:** endothelial glycocalyx, syndecan-1, congenital heart disease, cardiac surgery, pediatrics

## Abstract

**Background/Objectives**: In the present study, we hypothesized that cardiac surgery in pediatric patients with congenital heart disease (CHD) leads to profound endothelial glycocalyx degradation, measured as the increase in plasma syndecan-1 concentration, and that this endothelial damage is more pronounced in patients with cyanotic CHD. **Methods**: A total of 40 infants (24 with acyanotic and 16 with cyanotic CHD) were enrolled in this prospective study. A total of 39 cardiac surgeries were performed with cardiopulmonary bypass (CPB), 38 with CPB and aortic clamping, and 3 with CPB, aortic clamping, and deep hypothermic circulatory arrest. **Results**: Syndecan-1 concentrations increased significantly post-surgery compared to the baseline in both groups (cyanotic: 24.4 to 48.0 ng/mL, *p* < 0.0001; acyanotic: 28.8 to 59.8 ng/mL, *p* < 0.0001). However, there was no significant difference in syndecan-1 concentrations at any timepoint between children with cyanotic and those with acyanotic CHD. Baseline syndecan-1 showed no correlation with preoperative arterial oxygen saturation (r = 0.26, *p* = 0.102), hemoglobin (r = −0.3, *p* = 0.06), age (r = 0.15, *p* = 0.36), and weight (r = 0.13, *p* = 0.42). Of note, CPB time (r = 0.08, *p* = 0.63) and AC time (r = 0.03, *p* = 0.86) were not related to syndecan-1 concentrations at the end of surgery. **Conclusions**: Cardiac surgery leads to profound glycocalyx degradation in children with CHD detected by increased plasma syndecan-1 concentrations. Regardless of major pathophysiological differences, children with cyanotic and acyanotic CHD presented similar plasma syndecan-1 values throughout the study.

## 1. Introduction

Congenital heart disease (CHD) occurs in approximately 1% of live births and its etiology involves genetic, epigenetic, and environmental factors [1]. CHD can be subdivided into isolated or complex defects associated with syndromic conditions and into cyanotic (cCHD) and acyanotic (aCHD) defects based on oxygen saturation resulting from venous to arterial mixing. In pediatric patients with cCHD, the chronic hypoxemic state leads to complex and often severe alterations in whole-blood composition, viscosity, and coagulation profile [2]. Chronic hypoxemia is compensated for by an increase in erythropoietin production leading to secondary erythrocytosis, an increase in cardiac output, a release of nitric oxide, and a rightward shift in the oxygen dissociation curve. Endothelial prostaglandins and nitric oxide are released in response to increased shear stress, causing systemic arteriolar vasodilation. On the other hand, plasma endothelin-1 concentrations are elevated in children with cCHD. The combination of hypoxemia, elevated shear forces, and dysregulation of vasoactive mediators such as nitric oxide and endothelin-1 results in endothelial glycocalyx degradation and shedding of its constituent molecules into the circulation [3,4].

All human cells are coated by a surface layer of glycans called glycocalyx. The endothelial glycocalyx (EG) is a critical interface between the endothelium and the blood stream [5]. The composition of glycocalyx is in a state of constant flux, it continuously replenishes components that are removed by flowing plasma [6]. It has a highly complex and fragile structure consisting of various proteoglycans, glycosaminoglycans, and plasma proteins. It is an important regulator of vascular functions, such as transmission of shear stress, vascular tone and permeability, leukocyte recruitment, and coagulation. It plays a crucial role in vessel formation and maturation and is already present during early fetal development [5]. Dysregulated EG due to altered synthesis, shedding or intracellular degradation contributes to endothelial dysfunction in conditions such as systemic inflammation, sepsis, trauma, hypoxia, ischemia/reperfusion injury, and chronic inflammatory disorders including atherosclerosis and diabetes. The major components of EG, including syndecans, heparan sulphates, and hyaluronan, are shed under various clinical conditions. Increased EG shedding has been demonstrated during hypoxia, ischemia/reperfusion, inflammation, trauma, and sepsis in both pediatric and adult populations [4]. However, data on infants are still very limited. A few previous studies in pediatric cardiac surgery patients reported increased EG shedding, assessed as an increase in plasma syndecan-1 and/or hyaluronan concentrations, both of which are major components of EG [7,8]. These data provided the first evidence for basal turnover of vascular EG in pediatric cardiac surgery patients and showed that the shedding of EG increased with the extent of ischemia/reperfusion. However, none of these studies focused on patients with cyanotic CHDs.

In the present study, we hypothesized that (1) cardiac surgery in pediatric patients with CHD leads to profound glycocalyx degradation, reflected by an increase in plasma syndecan-1 concentrations, and that (2) this degradation is more pronounced in patients with cyanotic CHD than acyanotic CHD. To empirically investigate these hypotheses and further elucidate the impact of cardiac surgery on endothelial glycocalyx integrity in pediatric patients with CHD, we designed a prospective study as described in the following methods section.

## 2. Materials and Methods

The study was approved by the ethics committee of our institution (Ethics committee of the Medical University of Vienna, Austria; trial registration no. 1484/2019) and performed in accordance with the ethical standards laid down in the declaration of Helsinki. Written parental informed consent was obtained from each included patient.

Exclusion criteria were refusal of parental consent, life-threatening diseases apart from the underlying heart disease, emergency surgical procedures, preceding ICU stay (preoperative mechanical ventilation, mechanical circulatory support, or vasoactive medication), preoperative cardiopulmonary resuscitation, shock, and a preoperative exacerbation of inflammatory processes or clinical or laboratory signs of infection.

### 2.1. Anesthesia

Pediatric patients up to the 1st year of life received dexamethasone 1 μg/kg prior cardiac surgery per protocol. Tranexamic acid was administered to all patients undergoing cardiac surgery (15 mg/kg prior to the surgical procedure and a further 50 mg/kg added to the CPB priming volume) according to the local standard operating procedure.

Patients older than 6 months received intravenous midazolam (0.1 mg/kg) immediately prior to their transport to the operating room.

General anesthesia was induced with propofol, etomidate, midazolam, and/or ketamine, depending on the underlying disease, the overall constitution, hemodynamic stability, and the age of the child; fentanyl was used as the primary opioid, and rocuronium (0.6 mg/kg) was used as the primary neuromuscular blocking agent. Anesthesia was maintained either with midazolam or sevoflurane (1.5 to 2.5 vol.%) and fentanyl. Rocuronium was administered as bolus injections to provide sufficient neuromuscular relaxation.

Arterial and central venous catheters were placed after the induction of anesthesia. Intraoperative monitoring included end-tidal CO_2_, pulse oximetry, electrocardiogram, esophageal and rectal or bladder temperature measurement, diuresis monitoring, invasive arterial and central venous pressure measurement, as well as measurement of regional cerebral oxygenation using near-infrared spectroscopy.

### 2.2. Cardiopulmonary Bypass Protocol

Bypass circuits of the heart–lung machine were selected according to the calculated body surface area of the pediatric patient. Pump priming was performed according to local standard operating procedure. The bypass circuits were further primed with red blood cells to achieve a hematocrit of 30%.

### 2.3. Blood Sampling and Processing

Central venous blood samples were drawn after the induction of anesthesia before surgical incision (T0; defined as the baseline value) and at the end of the surgical procedure before admission to the intensive care unit (T1). Central venous blood sampling was chosen over arterial blood sampling to preserve the arterial line, which is known to be very fragile in small infants and neonates, for hemodynamic monitoring. Blood samples were processed immediately after collection, centrifuged at 2600× *g* for 15 min, and plasma aliquots were stored at −80 °C until analysis.

### 2.4. Quantification of Glycocalyx Shedding

Glycocalyx shedding was assessed using plasma syndecan-1 concentrations. Quantitative syndecan-1 levels were measured using a sandwich enzyme-linked immunosorbent assay, according to the manufacturer’s instructions (Diaclone Research; Besancon, France). Standards, samples, and controls were run in duplicate, and the resulting chromogen was read at 450 nm, with an additional wavelength correction at 570 nm. Syndecan-1 concentrations (ng/mL) were then calculated based on the constructed standard curves on respective enzyme-linked immunosorbent assay plates.

### 2.5. Statistical Analysis

Data were analyzed using the MedCalc Software (MeCalc Software bvba, Ostend, Belgium). Data are presented as medians and ranges. For paired data, comparisons were performed using the Wilcoxon signed-rank test. For unpaired data, comparisons were made using the Kruskal–Wallis test and the Mann–Whitney U test as appropriate. For the correlation between two variables, a Spearman correlation was calculated. *p* values < 0.05 were regarded as statistically significant.

## 3. Results

A total of 40 children with CHD undergoing cardiac repair surgery were enrolled in this prospective study, of which 24 children presented with aCHD and 16 had cCHD. A total of 39 cardiac surgeries were performed with cardiopulmonary bypass; 38 with CPB and aortic clamping; and 3 with CPB, aortic clamping, and deep hypothermic circulatory arrest. One surgery was performed without CPB. The baseline demographic and laboratory data for all patients as well as for the subgroups of patients with acyanotic and cyanotic CHD are depicted in Table 1.

There was a significant difference in age (cCHD median 3 months, range 0–75 months vs. aCHD median 12 months, range 0–140 months; *p* = 0.01) and weight (cCHD median 5.2 kg, range 3–17 kg vs. aCHD median 7.7 kg, range 3.8–22.7 kg; *p* = 0.01) between children with cyanotic versus acyanotic CHD. Of note for the interpretation of further results, children with cCHD were much younger and lighter at the time of cardiac surgery than children with aCHD. As expected, children with cCHD had a significantly lower arterial oxygen saturation than children with aCHD (median 85%, range 73–98% vs. median 98%, range 90–100%; *p* < 0.0001, respectively). Platelet counts and fibrinogen concentrations were not significantly different between children with cyanotic and acyanotic CHD. Although not statistically significant, hemoglobin concentrations were slightly higher in children with cCHD (median13.7 mg/dL, range 9.6–20.9) than in children with aCHD (median 11.3 mg/dL, range 9.8–21.8 mg/dL).

### 3.1. Syndecan-1 Shedding in Pediatric Cardiac Surgery

The absolute plasma syndecan-1 concentrations before and after pediatric cardiac repair surgery are shown in Table 2.

**Figure 1:**
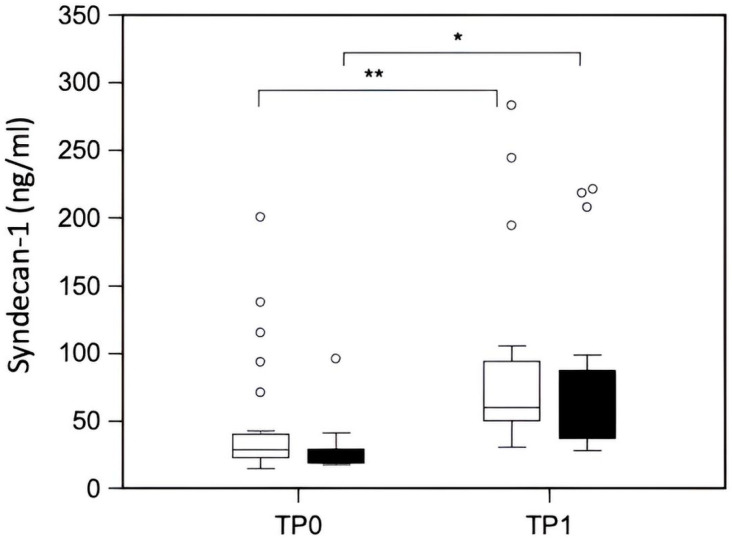
Plasma concentrations of syndecan-1 increases expressed as median in pediatric patients with aCHD (white bars) and cCHD (black bars). *, *p* < 0.05 and **, *p* < 0.05. Abbreviations: aCHD, acyanotic congenital heart disease; cCHD, cyanotic congenital heart disease; TP0, timepoint 0; TP1, timepoint 1. Hollow circle: mild Outliers.

There was a statistically significant increase in syndecan-1 levels after cardiac surgery (*p* < 0.0001). At the end of surgery, a 2.04-fold increase in syndecan-1 compared to the baseline was detected (Figure 1). No correlation has been observed between baseline syndecan-1 concentrations and preoperative arterial oxygen saturation (r = 0.26, *p* = 0.10), hemoglobin concentrations (r = −0.3, *p* = 0.06), age (r = 0.15, *p* = 0.36), and weight (r = 0.13, *p* = 0.42). Of note, CPB time (r = 0.08, *p* = 0.63) and AC time (r = 0.03, *p* = 0.86) were not related to syndecan-1 concentrations at TP1. Because of the small number of deep hypothermic cardiac arrests (n = 3), we did not calculate its effect on syndecan-1 concentrations.

### 3.2. Syndecan-1 Shedding in cCHD and aCHD

Absolute plasma syndecan-1 concentrations before and after pediatric cardiac repair surgery in children with cCHD and aCHD are shown in Table 2. There was a statistically significant increase in syndecan-1 concentrations after cardiac surgery in both cCHD (*p* < 0.0001) and aCHD (*p* > 0.0001) patients (Figure 1). At the end of surgery (TP1), we observed a 1.97-fold increase in syndecan-1 in cCHD and a 2.07-fold increase in syndecan-1 in aCHD compared to baseline values (TP0). However, there was no significant difference in syndecan-1 levels at any timepoint between children with cCHD and aCHD.

## 4. Discussion

In this prospective observational cohort study, we evaluated the endothelial biomarker syndecan-1 in cardiac repair surgery in children with a cyanotic and acyanotic CHD. We detected a significant increase in plasma syndecan-1 levels after cardiac surgery with cardiopulmonary bypass in all our pediatric patients. These findings align with previous studies in pediatric and adult populations and reflect the degradation of the endothelial glycocalyx [9,10] during cardiac surgery with cardiopulmonary bypass. Bruegger and coworkers showed a 3.0-fold increase in syndecan-1 concentration in infants undergoing cardiac surgery with CPB and aortic cross clamping [7]. They also described that pediatric CPB with combined ischemic challenge of aortic cross clamping and deep hypothermic circulatory arrest resulted in even higher plasma syndecan-1 concentrations than aortic cross clamping alone. However, based on results from another study, DHCA seems to not cause further shedding of glycocalyx components beyond the effects of CPB and aortic cross clamping [8]. Because only three patients in our study underwent DHCA additionally to CPB and aortic cross clamping, we have insufficient data to comment on this issue. Cardiopulmonary bypass is a known critical component of cardiac surgeries, as blood comes into contact with the artificial surfaces of the CPB circuit, causing additional stress and inflammatory reactions [11]. Direct contact of blood with CPB and heparin neutralization with protamine leads to activation of the complement cascade. The negative effects of CPB on glycocalyx degradation have been visualized previously by using side-stream dark field imaging showing a significantly increased perfused boundary region in 36 infants undergoing cardiac surgery with CPB, indicating reduced glycocalyx thickness due to shedding [10].

The endothelial glycocalyx is a complex and fragile endothelial layer consisting of glycoproteins and proteoglycans; it plays a major role in vascular hemostasis and permeability, vascular tone, coagulation, and interaction between the endothelium and leukocytes [5]. Systemic inflammation and consecutive release of reactive oxygen species (ROS) lead to endothelium damage and dysfunction. Various stimuli occurring during cardiac surgery such as surgical technique, shear stress, intravascular hypo- and hypervolemia, temperature changes, and drugs can trigger systemic inflammation and the release of ROS. One of the key mechanisms for syndecan-1 shedding is the activation of matrix metalloproteinases by pro-inflammatory cytokines and ROS. Previous studies have shown that matrix metalloproteinases are activated by ischemia–reperfusion injury and during cardiac surgery [12,13].

Furthermore, in the present study, plasma syndecan-1 concentrations were similar to those previously reported in pediatric patients undergoing cardiac surgical procedures [7,8,14]. In line with these results, we also observed a two-fold increase in syndecan-1 concentration after CPB in our study population. The consistency of our results with published data is particularly notable given the current lack of pediatric reference values and the ongoing challenges in interpreting glycocalyx markers across different age groups [5]. While most research regarding the EG was performed in adult populations, data on development of glycocalyx and its involvement in physio- and pathological conditions in pediatric populations is limited. More data on EG in pediatric patients are needed to differentiate possible age-dependent differences on the shedding and regeneration of EG between pediatric and adult patients.

Due to chronic hypoxemia, pediatric patients with cCHD suffer from endothelial, hematologic, and hemostatic alterations [2]. In the present study, at the time of cardiac surgery, pediatric patients with cCHD had a statistically significant, lower age and body weight compared to patients with aCHD. Furthermore, patients with cCHD had statistically significant, lower SaO_2_ levels and slightly, but statistically not significant, higher hemoglobin concentrations than patients with aCHD. Despite these clinically relevant findings, we found no statistically significant difference in plasma syndecan-1 concentrations between children with cCHD and those with aCHD at any timepoint. This result was unexpected, because hypoxia alone is sufficient to produce glycocalyx loss, and experimental studies focusing on hypoxia have shown impaired endothelial function such as increased permeability and disturbed endothelium-dependent vasodilator responsiveness of resistance vessels [15,16]. Furthermore, secondary erythrocytosis in response to hypoxemia increases whole-blood viscosity and shear stress [3]. This leads to an increased release of endothelial prostaglandins, nitric oxide, and reduced vascular tone [17]. Furthermore, the adaptive response to hypoxia is primarily mediated by the hypoxia-inducible transcription factor HIF-1, which leads to the induction of a variety of adaptive gene products [18]. As previously described by Quing and colleagues, HIF-1 activity is elevated in the myocardium of infants with cCHD compared to those with aCHD, and this response directly correlates with the degree of hypoxemia [7]. HIF-1 mediates the induction of vascular endothelial growth factor (VEGF) and endothelial nitric oxide synthase. This mechanism may play an important role in neovascularization, the formation of abnormal vessels, and cardiac remodeling seen in patients with cCHD. Nevertheless, components of glycocalyx have also been shown to be critically involved in angiogenesis [19,20,21]. All these results would suggest higher turnover of glycocalyx in pediatric patients with cCHD. However, faster regeneration or steady state of EG in pediatric patients may affect our results. Previous studies have shown a significant reduction in glycocalyx thickness, assessed by an increase in perfused boundary region (PBR) using side-stream dark field imaging, in both pediatric and adult patients after cardiac surgery with cardiopulmonary bypass [10,22,23]. However, the thickness of EG started to increase after 24 h in pediatric patients. In contrast, in adult populations, the PBR was still decreasing for the next 3 postoperative days. Due to limited data on the regeneration of glycocalyx in children, we can only speculate that the lack of difference in plasma syndecan-1 levels between cCHD and aCHD patients at baseline may reflect faster regeneration than in adult patients. The age-dependent difference in glycocalyx shedding has also been observed in a clinical trial, where a high dose of methylprednisolone reduced glycocalyx damage in neonates, but not in older infants, undergoing cardiac surgery [8]. Although we expected a more fragile glycocalyx in patients with cCHD than with aCHD due to chronic hypoxia, our results did not support this hypothesis. The difference in increase in syndecan-1 between the groups was clinically irrelevant. Measuring syndecan-1 at only two timepoints did not allow us to perform more extensive analyses on the kinetics of syndecan-1. Further studies including measurements at multiple timepoints may elucidate kinetics of syndecan-1 in this patient population. Better understanding of EG degradation as a mechanism of endothelial dysfunction is needed to improve patient management during and after cardiac surgery. The interplay of major inflammatory response and oxidative stress occurring during cardiac surgery with EG is complex and still not fully understood. Further research, particularly in pediatric patients, is needed to develop effective strategies to enhance regeneration and improve outcome.

Our study has several limitations. First, the observational design only allows the description of associations rather than causations regarding surgical factors and glycocalyx degradation in pediatric patients with aCHD and cCHD. Second, the heterogeneity of cardiac defects and surgical procedures, may have introduced unmeasured confounding, though these are inherently linked to age and diagnosis. Third, age and weight differences between groups complicate direct comparisons, though these disparities reflect real-world clinical practice patterns. Fourth, we measured only syndecan-1; additional glycocalyx markers (hyaluronan, heparan sulfate) would provide a more comprehensive assessment. Fifth, single postoperative measurement prevents evaluation of recovery kinetics.

We conclude that there is significant glycocalyx degradation in pediatric patients with congenital heart disease, detected as an increase in plasma syndecan-1 concentrations after cardiac surgery. Regardless of major pathophysiological differences, pediatric patients with cCHD and those with aCHD presented with similar plasma syndecan-1 values throughout the study. The absence of between-group differences, despite major pathophysiological disparities, suggests either enhanced glycocalyx regenerative capacity in pediatric populations, masking chronic injury or acute surgical trauma dominating over chronic pre-existing endothelial stress. Further studies are needed to fully understand the underlying mechanisms and their clinical relevance in this vulnerable pediatric population.

## Figures and Tables

**Table 1:** Patient demographic, baseline laboratory, and procedural characteristics.

	All (n = 40)	cCHD (n = 16)	aCHD (n = 24)	*p*
Age (months)	7 (0–140)	3 (0–75)	12 (0–140)	0.016
Weight (kg)	6.5 (3–22)	5.2 (3.0–17.0)	7.7 (3.8–22.7)	0.01
Male/female (n)	24/16	8/8	16/8	0.33
SaO_2_ (%)	96 (73–100)	85 (73–98)	98 (90–100)	<0.0001
Hb (g/dL)	11.7 (9.6–21.8)	13.7 (9.6–20.9)	11.3 (9.8–21.8)	0.05
WBC (G/L)	9.0 (4.3–16.9)	9.7 (7.5–15.3)	8.2 (4.3–16.9)	0.01
PLT (G/L)	348 (116–600)	348 (224–600)	347 (116–560)	0.66
Fib (mg/dL)	230 (180–400)	237 (182–344)	230 (180–400)	0.70
Crea (mg/dL)	0.3 (0.15–0.86)	0.35 (0.15–0.86)	0.3 (0.15–0.58)	0.18
CPB (min)	104 (24–321)	136 (24–321)	97 (38–252)	0.07
ACC (min)	66.5 (0–224)	68 (0–149)	65 (19–224)	0.62

Abbreviations: ACC, aortic cross clamp; aCHD, acyanotic congenital heart disease; cCHD, cyanotic congenital heart disease; CPB, cardiopulmonary bypass; Crea, serum creatinine concentration; Fib, fibrinogen concentration; Hb, hemoglobin concentration; PLT, platelet count; SaO_2_, arterial oxygen saturation; WBC, white blood cell count.

**Table 2:** Plasma concentrations of syndecan-1 (ng/mL) in median (ranges).

	TP0	TP1	*p*
All (n = 40)	27.6 (814.4-200.7)	56.3 (28.6–283.5)	0.0001
cCHD (n = 16)	24.4 (17.9–96.3)	48.0 (28.6–221.3)	0.0001
aCHD (n = 24)	28.8 (14.7–200.7)	59.8 (30.7–283.5)	0.0001

Abbreviations: aCHD, acyanotic congenital heart disease; cCHD, cyanotic congenital heart disease, TP0, timepoint 0; TP1, timepoint 1. There was a statistically significant increase in syndecan-1 concentration at postoperative timepoint 1 (TP1) compared to the preoperative value (TP0) in each group.

## Data Availability

The data relevant to this study cannot be made publicly available, as it contains sensitive patient information concerning a vulnerable population (infants under 1 year of age). Access to the data is restricted by the Data Access Committee of the Medical University of Vienna in accordance with Article 9 of the General Data Protection Regulation (GDPR). For inquiries or data access requests, please contact: datenclearing@meduniwien.ac.at (https://www.meduniwien.ac.at/web/en/about-us/organisation/committees/data-clearing-house/?L=3RO).

## Abbreviations

The following abbreviations are used in this manuscript:

aCHD	Acyanotic congenital heart disease
CHD	Congenital heart disease
cCHD	Cyanotic congenital heart disease
EG	Endothelial glycocalyx
ROS	Reactive oxygen species
